# Social defeat predicts the emergence of psychotic-like experiences through the effects on aberrant salience: insights from a network analysis of longitudinal data

**DOI:** 10.1017/S0033291724003209

**Published:** 2024-12

**Authors:** Tomasz Bielawski, Maksymilian Rejek, Błażej Misiak

**Affiliations:** Department of Psychiatry, Wroclaw Medical University, Pasteura 10 Street, 50-367 Wroclaw, Poland

**Keywords:** depression, humiliation, psychosis, schizophrenia, social defeat

## Abstract

**Background:**

Psychotic-like experiences (PLEs) are subclinical phenomena that often precede the onset of psychosis and occur in various mental disorders. Social determinants of psychosis and PLEs are important and have been operationalized within the social defeat (SD) hypothesis. The SD hypothesis posits that low social status and exposure to repeated humiliation can lead to imbalanced dopamine neuron activity, and thus increased risk of psychosis. We aimed to assess the role of dynamic interactions between SD components in shaping the occurrence of PLEs using a network analysis.

**Methods:**

A total of 2241 non-clinical, young adults were assessed at baseline and invited for reassessment after a 6-month follow-up. Self-reports recording the occurrence of PLEs, aberrant salience (AS), depressive, and anxiety symptoms as well as SD characteristics (socioeconomic status, minority status, humiliation, perceived constraints, and domain control) were administered. Two networks were analyzed (the first one covering all baseline measures and the second one with the baseline SD components and follow-up measures of AS and psychopathology).

**Results:**

The SD components were not directly connected to the measures of PLEs in both networks. However, in both networks, SD components were connected to PLEs through a mediating effect of AS. Among SD components, humiliation had the highest bridge centrality across three predefined communities of variables (SD; depressive and anxiety symptoms; AS, and PLEs).

**Conclusions:**

The findings indicate that SD might make individuals vulnerable to develop PLEs through the mediating effects of AS. Among SD components, humiliation might play the most important role in the development of PLEs.

## Introduction

Psychotic-like experiences (PLEs) capture subclinical phenomena that affect up to 7% of the general population (McGrath et al., 2015). Studies have shown that PLEs are a transdiagnostic risk factor for multiple mental disorders, including psychosis, bipolar disorder, and major depression (Linscott & van Os, 2013; Pain et al., 2018). In recent years, a notable shift from investigating categorical diagnostic constructs towards dimensional approaches can be observed, and PLEs constitute an important dimension of symptoms that share etiological risk factors with psychosis (van Os, Linscott, Myin-Germeys, Delespaul, & Krabbendam, 2009), and might precede the onset of psychotic disorder (Kaymaz et al., 2012). Importantly, PLEs develop as the consequence of multiple psychological processes including, i.e. psychosocial stress, a history of childhood trauma, and cognitive biases (Linscott & van Os, 2013). Aberrant salience (AS) is an important example of the latter – a phenomenon of excessive assignment of significance and relevance to irrelevant environmental stimuli, observed during the prodromal phase of psychosis (Howes, Hird, Adams, Corlett, & McGuire, 2020). Recent literature posits AS as an important phenomenon that fits into the psychosis risk continuum (Reininghaus et al., 2016). Moreover, there is a growing body of evidence suggesting shared neurobiological backgrounds of AS and schizophrenia in terms of deregulated dopaminergic and glutaminergic pathways (Howes et al., 2020; Panayi et al., 2023).

To date, a number of various risk factors have been associated with the development of psychotic disorders including genetic factors (Legge et al., 2021), urbanicity (Vassos, Pedersen, Murray, Collier, & Lewis, 2012), obstetric complications (Sagué-Vilavella et al., 2022), substance abuse (Khokhar, Dwiel, Henricks, Doucette, & Green, 2017), and psychosocial stress (Selten & Cantor-Graae, 2005). The social defeat (SD) hypothesis suggests that individuals who experience prolonged subordinate social status, repeated humiliation, or exclusion from the majority group are at an elevated risk of developing psychosis. This is thought to alter homeostatic control of dopaminergic neurons in the midbrain and dorsal striatum, which might lead to increased striatal dopamine activity (Selten & Cantor-Graae, 2005; Selten, Van Der Ven, & Termorshuizen, 2020). The SD hypothesis of psychosis has been widely discussed in the context of increased risk of psychotic disorders in migrants, disadvantaged ethnic minorities, and individuals with persistent low status (Selten et al., 2020; van Nierop et al., 2014). Although the SD hypothesis of psychosis has attracted research activity for more than a decade, it still receives a fair amount of critique, mainly due to a lack of direct, easily measurable indicators useful in empirical research (Fletcher & Birk, 2022; Schalbroeck, 2023).

Importantly, SD might be associated with a number of psychological processes being the consequence of low social status that have not been thoroughly investigated so far. One of them is the experience of cumulative humiliation defined as repeated, unresolved experiences of disconnection from relations with others (Hartling & Luchetta, 1999), often described in the context of socially rejected groups (Hanna et al., 2019; Selten et al., 2020). It might also be operationalized as an effect of interpersonal dynamics, where individuals are unable to maintain adequate levels of growth-fostering relationships with others (Hartling & Luchetta, 1999). Prolonged humiliation associated with increased dopamine levels and a higher risk of psychosis might be a central facet of the SD hypothesis (Isovich, Engelmann, Landgraf, & Fuchs, 2001; Selten, van der Ven, Rutten, & Cantor-Graae, 2013; Selten et al., 2020). Humiliation might also be closely related to ‘domain of control’ and ‘perceived constrains’ – two indicators of an individual's feeling of authority over the results of their own actions, as well as their views on their position in different areas of life activities, such as work, family, and even broader social environments. Both domain control and perceived constraints are hypothesized to reflect perceived status among close ones, often referred to as the status syndrome (Seeman, Stein Merkin, Karlamangla, Koretz, & Seeman, 2014). The core of the status syndrome lies in one's sense of control and perceptions of their social standing across various life domains and social hierarchies (Marmot, 2004; Seeman et al., 2014). Low status syndrome is often associated with a broader biological dysregulation leading to poorer overall health and higher rates of clinically relevant morbidities (Seeman et al., 2014). Interestingly, similar indices (low socioeconomic status and limited social network size) have also been associated with psychosis risk (Jester et al., 2023).

Minority status is another SD component as being a part of a socially excluded group is often linked with numerous adversities and social isolation (Hanna et al., 2019; Selten et al., 2020). The SD hypothesis also needs to be considered in light of individual average income, employment status, and the level of education, commonly referred to as the indices of socioeconomic status (Cooper, 2005). Lower socioeconomic status, experiences of discrimination and isolation, and limited social network have been widely associated with psychosis risk (Goldberg & Morrison, 1963; Jester et al., 2023).

In light of these considerations, there are various components of the SD hypothesis. However, the role of their mutual interactions in shaping PLEs have not been thoroughly investigated so far. Recent advances in analytical approaches to psychopathology have provided a novel tool known as a network analysis (Borsboom et al., 2021). This approach offers testing multiple variables in a single model without a predefined direction of causality. Taking into consideration existing research gaps in the field, the present study aimed to investigate dynamic interactions of SD components, including socioeconomic status, minority status, humiliation, domain control, and perceived constraints with AS and PLEs in a non-clinical sample of young adults.

## Material and methods

### Participants

The study was based on an online survey. Invitations were sent through the online platform designed for research surveys. Recruitment procedures were implemented in December 2023 and the survey was conducted using computer-assisted web interviews. The participants were recruited from the online panel representative of the Polish population and maintained by a research company. All of them received compensation for participation (equivalent to 10 EUR). The inclusion criteria were age between 18 and 40 years and a lack of prior history of psychiatric treatment. Additionally, participants were selected to reflect the socio demographic characteristics of the Polish population based on data from 2021. Participants were reassessed after 6 months (June 2024). They were assessed with respect to the SD components (at baseline) as well as psychopathological symptoms and AS (at baseline and after 6 months). All participants agreed to participate in an online survey. The study obtained approval from the Bioethics Committee at Wroclaw Medical University, Wroclaw Poland (approval number: 22/2024).

### SD components

#### Humiliation

We used the Humiliation Inventory to assess the internal experience of humiliation (Hartling & Luchetta, 1999). The inventory consists of 32 self-reported items rated between 1 (‘not harmed at all’) and 5 (‘extremely harmed’). The questionnaire in its original form includes two subscales, i.e. the cumulative humiliation subscale and the fear of humiliation subscale. The first one measures the severity of past humiliating experiences, while the latter one records the level of anticipation and anxiety regarding future humiliating experiences. In our study, we used the first subscale only that is based on 12 items and aims to measure lifetime cumulative humiliation. The total score ranges between 12 and 60 where higher scores reflect higher levels of humiliation experiences. The Cronbach's alpha was 0.961 in the present study.

#### Perceived constraints and domain of control

We used the measures developed by Seeman et al. (2014), which build upon the concepts initially described by Lachman and Weaver (1998) to assess individual sense of control in the social environment. The first one was the Perceived Constraints Scale (8 items, e.g. ‘I often feel helpless in dealing with the problems of life’ and ‘Other people determine most of what I can and cannot do’) with each item rated on a 7-point scale (from 1 – ‘strongly agree’ to 7 – ‘strongly disagree’). The total score ranges between 8 and 56 with lower scores representing higher levels of perceived constraints. The second one was the Domain of Control Scale that includes 5 items (e.g. ‘Please rate the amount of control you have over: your work situation these days; your financial situation these days; your contribution to the welfare and well-being of other people these days’) rated on a 10-point scale (from 1 – ‘total lack of control’ to 10 – ‘complete control’). The total score ranges between 10 and 50, where higher scores are equivalent to higher levels of domain control. The Cronbach's alpha values for the Perceived Constraints Scale and Domain of Control Scale were 0.893 and 0.815, respectively.

#### Other SD measures

Participants were asked about the level of education, the current vocational situation, an average income per month, and perceived minority status. The level of education was categorized as follows: (1) primary (graduation from primary school only), (2) vocational (gives preparation for professional employment, based on education in basic vocational schools, basic schools or other equivalent schools, or study of crafts); (3) secondary (graduation from a general or vocational secondary school), and (4) higher (obtaining a bachelor's, engineer's, master's, or equivalent degree; this category also included the PhD degree). The current vocational situation was categorized as unemployed (i.e. a lack of employment, or student status), student (in the course of studies, i.e. the first, second, or third degree without full-time job), employed (part-time or full-time job). The average income per month was categorized by multiplying the minimum wage value identified in 2023 in Poland.

The perceived minority status was assessed using a single item: ‘Do you feel a part of the minority: (1) sexual minority; (2) religious minority; (3) cultural minority;or (4) none?’.

#### Aberrant salience

The Aberrant Salience Inventory (ASI) was used to measure the tendency to assign meaning to irrelevant stimuli. The ASI consists of 29 self-report items with a yes-or-no scoring (rated as 1 or 0). It is based on five subscales (increased significance, senses sharpening, impending understanding, heightened emotionality, and heightened cognition) and has good psychometric properties (Cicero, Kerns, & McCarthy, 2010). Several studies have confirmed that the ASI is related to a higher risk of psychosis (Howes et al., 2020; Reininghaus et al., 2016). The Cronbach's alpha for ASI was 0.925 in the present study.

#### PLEs

We used the Prodromal Questionnaire-16 (PQ-16) to record the presence of PLEs over the preceding month (Ising et al., 2012). The PQ-16 has been designed to detect psychosis risk states. It consists of 16 true-or-false items that capture various PLEs, along with associated distress rated from 0 (lack of distress) do 4 (significant distress). Two items (i.e. items 1 and 7) might measure depressive and anxiety symptoms. Therefore, we analyzed 14 remaining items with the total score ranging between 0 and 14. In the present study, the Cronbach's alpha value for the presence subscale was found to be 0.844. In turn, the Cronbach's alpha for the distress subscale was 0.859.

#### Depressive and anxiety symptoms

Due to the fact that PLEs are widely perceived as transdiagnostic phenomena, the present study also recorded the occurrence of depressive and anxiety symptoms. To assess the levels of depressive symptoms, the Patient Health Questionnaire-9 (PHQ-9) was administered (Kroenke, Spitzer, & Williams, 2001). It records the level of depressive symptoms experienced over the preceding two weeks, using a four-point scale. Responses to each item range from 0 – ‘not at all’ to 3 – ‘nearly every day’. The overall score ranges between 0 and 27 (higher scores correspond with greater levels of depressive symptoms). In this study, the Cronbach's alpha of the PHQ-9 was 0.875.

To record the levels of anxiety symptoms, the Generalized Anxiety Disorder-7 (GAD-7) was used (Linscott & van Os, 2013). It records the severity of anxiety symptoms experienced over the preceding two weeks. Responses to each item range from 0 – ‘not at all’ to 3 – ‘nearly every day’. The total score ranges between 0 and 21 (higher scores corresponded with greater levels of anxiety symptoms). In this study, the Cronbach's alpha of the GAD-7 was 0.915

### Data analysis

There was no missing data in the present dataset, except for those related to monthly income status (Table 1). The general characteristics of participants who completed both assessments (further referred to as completers) and those who were lost to follow-up (further referred to as non-completers) were compared using the Mann–Whitney *U* test or *t* tests (in case of continuous variables depending on data distribution) and the c^2^ tests (in case of categorical variables). The level of significance was set at *p* < 0.05. These analyses were carried out in the SPSS software, version 28. Next, two partial correlation networks were estimated using the EBICglasso algorithm (Epskamp, Borsboom, & Fried, 2018). The first one was limited to baseline data while the second one included SD components together with follow-up measures of AS and psychopathology. The EBICglasso algorithm is based on the use of the Least Absolute Shrinkage and Selection Operator (LASSO) to regularize the network (Foygel & Drton, 2010). The LASSO shrinks small correlation coefficients to zero values to avoid indicating weak associations. Therefore, only significant associations are visualized in the network. The resulting network shows included variables that are presented as nodes connected with edges. Thicker and more saturated edges correspond with stronger associations. To assess the importance of specific nodes, the bridge expected influence was analyzed (Jones, Ma, & McNally, 2021).

**Table 1.** Descriptive characteristics of the sample

	Completers, *n* = 1308	Non-completers, *n* = 933	*p*
Age, years	31.1 ± 5.9	29.1 ± 6.6	<0.001
Gender			
Men	627 (47.9)	413 (44.3)	0.049
Women	677 (51.8)	520 (55.7)	
Other	4 (0.3)	0 (0)	
Education			
Primary	23 (1.8)	41 (4.4)	<0.001
Vocational	75 (5.7)	86 (9.2)	
Secondary	489 (37.4)	419 (44.9)	
Higher	721 (55.1)	387 (41.5)	
Unemployment, yes	153 (11.7)	146 (15.6)	0.008
Income^a^			
<750 USD	317 (28.0)	234 (31.5)	0.218
750–1500 USD	605 (53.5)	391 (52.6)	
1500–2500 USD	174 (15.4)	93 (12.5)	
2500–3750 USD	27 (2.4)	17 (2.3)	
>3750 USD	8 (0.7)	9 (1.2)	
Minority status, yes	310 (23.7)	248 (26.6)	0.120
Humiliation	27.0 ± 12.2	27.0 ± 12.1	1.000
Domain control	35.6 ± 10.9	35.8 ± 11.1	0.616
Perceived constraints	30.0 ± 9.6	29.5 ± 9.7	0.279
Aberrant salience, baseline	9.4 ± 7.7	10.4 ± 7.3	0.002
Aberrant salience, follow-up	9.0 ± 7.9	–	–
Depressive symptoms, baseline	7.9 ± 5.7	8.4 ± 5.5	0.059
Depressive symptoms, follow-up	7.7 ± 5.8	–	–
Anxiety symptoms, baseline	7.2 ± 5.2	7.6 ± 5.1	0.150
Anxiety symptoms, follow-up	7.1 ± 5.3	–	–
PLEs – presence, baseline	3.2 ± 3.4	3.7 ± 3.3	<0.001
PLEs – presence, follow-up	3.0 ± 3.5	–	–
PLEs – distress, baseline	4.4 ± 5.9	5.4 ± 6.3	<0.001
PLEs – distress, follow-up	4.3 ± 6.1	–	–

Data are shown as mean ± s.d. or *n* (%).

a Missing data in case of 177 completers and 189 non-completers.

PLEs, psychotic-like experiences.

In the next step, node centralities were assessed. In general, centrality shows the importance of nodes in the network by estimating their overall strength of connections with other nodes. In the case of networks, where the communities of nodes differ with respect to their number, some nodes might show inflated centrality due to the high number of strong connections within the community. For instance, in our study, the number of nodes representing SD components (*n* = 7) was higher than the number of nodes representing psychopathology (*n* = 3). Therefore, we considered the inclusion of bridge centrality metrics (Jones et al., 2021). Specifically, we decided to assess the bridge expected influence. It shows the total weight of connections between a specific node with all nodes representing other communities. As opposed to other centrality metrics, it considers the presence of negative edges (Robinaugh, Millner, & McNally, 2016). The 1-step bridge expected influence shows the extent of direct connections, while the 2-step bridge expected influence also considers in direct connections (i.e. through mediating effects of various nodes). Before performing the analysis of bridge expected influence, three communities of nodes were predefined: (1) the SD components (socioeconomic status nodes, minority status, humiliation, domain control, and perceived constraints); (2) depressive and anxiety symptoms, and (3) AS and PLEs.

Finally, the network stability was assessed through bootstrapping procedures with 1000 iterations. The network was interpreted as stable if the correlation stability coefficient (CS-C) was higher than 0.25 (ideally it should be higher than 0.50) (Epskamp et al., 2018). All analyses were carried out in the R software (version 4.1.3) using the following packages: *networktools* (Jones et al., 2021), *bootnet* (Epskamp et al., 2018), *qgraph* (Epskamp, Cramer, Waldorp, Schmittmann, & Borsboom, 2012), and *mgm* (Haslbeck & Waldorp, 2020).

## Results

A total of 4756 participants were approached for participation. Among them, 1098 individuals (23.1%) declared a positive lifetime history of psychiatric treatment and 1417 individuals declined to participate in the survey or were unresponsive (29.8%). Finally, 2241 individuals (30.3 ± 6.3 years, 53.4% females) were surveyed (Table 1). The participants were most likely to report a higher education level (49.4%), full-time work status (66.7%), and a monthly income equivalent to 750–1500 USD (53.1%). The minority status was reported by 24.9% participants. Non-completers were younger, had lower education levels, and were more likely to be unemployed (*p* < 0.05). They had significantly higher baseline levels of PLEs (in terms of their presence and associated distress) and aberrant salience. Other significant differences between completers and non-completers were not observed.

**Figure 1.**
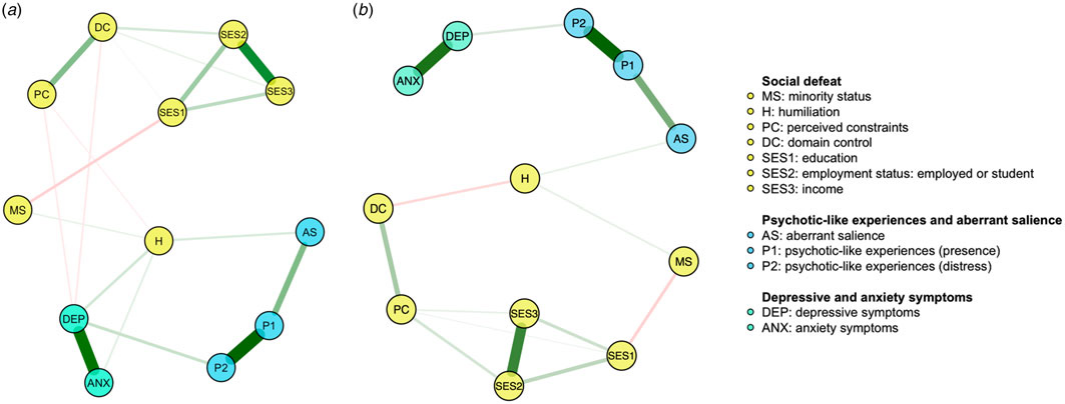
The networks analyzed in the present study (a – the network of baseline measures; b – the network of baseline social defeat components and follow-up measures of aberrant salience and psychopathology). Specific variables are shown as nodes connected with edges. Thicker and more saturated edges correspond with stronger associations. All of them indicate significant associations. Green edges represent positive correlations while red edges show negative correlations.

The network of baseline measures is shown in Fig. 1a. All nodes were well-connected and 19 edges (out of 66 potential edges, 28.8%) had a non-zero weight (Table 2). The nodes representing SD were not directly connected to the nodes of PLEs. However, two pathways linking SD with PLEs were identified. In the first pathway, the SD components appeared to be associated with PLEs through the mediating effect of AS. Indeed, humiliation was directly connected to the node of AS (weight = 0.098). In turn, the node of AS was directly connected to the node representing the occurrence of PLEs (weight = 0.333). Also, a greater level of humiliation was directly connected to higher levels of perceived constraints (weight = −0.041), depressive symptoms (weight = 0.119), and anxiety symptoms (weight = 0.070) as well as the perceived minority status (weight = 0.055). The second pathway linking SD components with PLEs led through the mediating effect of depressive symptoms. Indeed, depressive symptoms were directly connected to humiliation (weight = 0.119), perceived constraints (weight = −0.058), domain control (weight = −0.062), and the level of distress associated with PLEs (weight = 0.145).

**Table 2.** Edge weights in the network of baseline measures

	MS	H	AS	PC	DC	P1	P2	DEP	ANX	SES1	SES2
H	0.055	–									
AS	0.000	0.098	–								
PC	0.000	−0.041	0.000	–							
DC	0.000	0.000	0.000	0.327	–						
P1	0.000	0.000	0.333	0.000	0.000	–					
P2	0.000	0.000	0.000	0.000	0.000	0.753	–				
DEP	0.000	0.119	0.000	−0.058	−0.062	0.000	0.145	–			
ANX	0.000	0.070	0.000	0.000	0.000	0.000	0.000	0.684	–		
SES1	−0.126	0.000	0.000	0.000	0.020	0.000	0.000	0.000	0.000	–	
SES2	0.000	0.000	0.000	0.000	0.110	0.000	0.000	0.000	0.000	0.256	–
SES3	0.000	0.000	0.000	0.000	0.053	0.000	0.000	0.000	0.000	0.174	0.574

ANX, anxiety symptoms; AS, aberrant salience; DEP, depressive symptoms; DC, domain control; H, humiliation; MS, minority status; P1, psychotic-like experiences (presence); P2, psychotic-like experiences (distress); PC, perceived constraints; SES1, socioeconomic status (education); SES2, socioeconomic status (employed or student); SES3, socioeconomic status (income).

The network of baseline SD components and follow-up measures of psychopathology and AS is shown in Fig. 1b. All nodes were well-connected and 15 edges (out of 66 potential edges, 22.7%) had a non-zero weight (Table 3).The nodes representing SD were not directly connected to the nodes of PLEs. As similar to the network of with baseline measures, the nodes of SD were connected to the of PLEs through the mediating effects of AS. Specifically, humiliation was directly connected to the node of AS (weight = 0.068). In turn, the node of AS was directly connected to the node representing the occurrence of PLEs (weight = 0.416). A greater level of humiliation was associated with minority status (weight = 0.072) and a lower level of domain control (weight = −0.117). As opposed to the network of baseline measures, the mediating effect of depressive symptoms was not observed.

**Table 3.** Edge weights in the network of social defeat and follow-up measures of psychopathology and aberrant salience

	MS	H	AS	PC	DC	P1	P2	DEP	ANX	SES1	SES2
MS	–										
H	0.072	–									
AS	0.000	0.068	–								
PC	0.000	0.000	0.000	–							
DC	0.000	−0.117	0.000	0.273	–						
P1	0.000	0.000	0.416	0.000	0.000	–					
P2	0.000	0.000	0.000	0.000	0.000	0.791	–				
DEP	0.000	0.000	0.000	0.000	0.000	0.000	0.110	–			
ANX	0.000	0.000	0.000	0.000	0.000	0.000	0.000	0.714	–		
SES1	−0.140	0.000	0.000	0.039	0.000	0.000	0.000	0.000	0.000	–	
SES2	0.000	0.000	0.000	0.130	0.000	0.000	0.000	0.000	0.000	0.203	–
SES3	0.000	0.000	0.000	0.064	0.000	0.000	0.000	0.000	0.000	0.151	0.621

ANX, anxiety symptoms; AS, aberrant salience; DEP, depressive symptoms; DC, domain control; H, humiliation; MS, minority status; P1, psychotic-like experiences (presence); P2, psychotic-like experiences (distress); PC, perceived constraints; SES1, socioeconomic status (education); SES2, socioeconomic status (employed or student); SES3, socioeconomic status (income).

**Figure 2.**
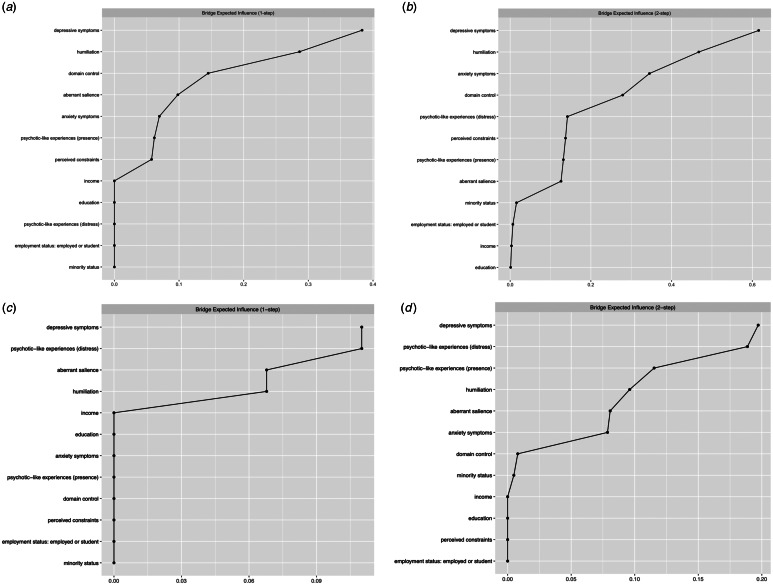
The bridge expected influence centrality. (a and b) Show results for the network of baseline measures while c and d show results for the network of social defeat components and follow-up measures of aberrant salience and psychopathology. Specific variables are ranked from the highest centrality (top part of the plot) to the lowest centrality (bottom part of the plot) in the network.

The bridge expected Influence is illustrated in Fig. 2. In the network limited to baseline measures, three nodes with the highest bridge expected influence were depressive symptoms, humiliation, and domain control (1-step bridge expected influence) or anxiety symptoms (2-step bridge expected influence). In turn, in the network with baseline SD components and follow-up measures of psychopathology and AS, depressive symptoms, the level of distress related to PLEs, and AS (1-step bridge expected influence) or the level PLEs occurrence (2-step bridge expected influence) were the three nodes with the highest bridge expected influence. In both networks, humiliation was the SD component with the highest bridge expected influence.

The network had sufficient stability while dropping various proportions of data. The CS-C values for edge weights and the bridge expected influence in a network analysis of baseline data were 0.75 and 0.52, respectively. In turn, the CS-C values in a network with follow-up measures of psychopathology and AS were 0.75 (for edges) and 0.44 (for bridge expected influence).

## Discussion

The main findings of the present study suggest that there is no direct connection between SD components and PLEs. However, we found that aberrant salience and depressive symptoms serve as the variables that might fully mediate the association between SD and PLEs. However, with respect to depressive symptoms, their mediating effect was only observed within cross-sectional data. Moreover, humiliation appeared to be the SD component that might be most closely related to PLEs.

To the best of our knowledge, this is the first study exploring the association between SD and AS. In both networks, AS mediated the association between SD components and PLEs. Several studies have indicated an association between social status, heightened awareness (paranoia), and the development of psychosis (Bentall, Wickham, Shevlin, & Varese, 2012; Gilbert, Boxall, Cheung, & Irons, 2005). Both AS and paranoid ideation refer to the concept of heightened sensitivity to neutral stimuli that creates a self-referencing bias (Fenigstein & Vanable, 1992). Prolonged exposure to victimization and other social adversities has been associated with paranoia in the general population (Bentall et al., 2012). Interestingly, one study based on a virtual reality environment revealed that individuals at high risk of psychosis, with a history of SD, present an increased tendency to make paranoid appraisals of social situations (Valmaggia et al., 2015). Moreover, a lower social rank has also been linked to paranoid thinking in healthy individuals and in early psychosis (Allison, Harrop, & Ellett, 2013; Gilbert et al., 2005). Our findings are in agreement with these observations as we found that AS appeared to be associated with humiliation.

Our study also demonstrated the presence of an alternative pathway between SD and PLEs, i.e. through the mediating effect of depressive and anxiety symptoms. However, this pathway was observed only in the network analysis limited to baseline measures. The failure to replicate this observation in the network with follow-up measures of aberrant salience and PLEs might be due to significant differences between completers and non-completers. Indeed, non-completers had significantly higher baseline levels of AS and PLEs. Both groups also differed significantly in terms of age, gender, education, and employment status. In this regard, non-completers might be considered the group with a greater vulnerability to develop clinically relevant psychopathology. However, completers and non-completers did not differ significantly in terms of baseline levels of depressive and anxiety symptoms. A recent systematic review and meta-analysis showed a mediating role of affective symptoms in the pathway between traumatic experiences and PLEs (Alameda et al., 2020). Affective symptoms have also been conceptualized in terms of the ‘affective pathway’ that leads to the development of PLEs, and the emergence of a reactive phenotype of psychosis (Myin-Germeys & Van Os, 2007). Our results are in line with those from a recent study linking PLEs with depression and anxiety (Cowan & Mittal, 2021), both of which have been described as mediators between personality traits and the occurrence of PLEs (Prochwicz & Gawęda, 2016). Other reports have revealed that anxiety and depression are associated with transition to the prodromal phase of psychosis in the group of young adults (Fusar-Poli, Nelson, Valmaggia, Yung, & McGuire, 2014; Varghese et al., 2011).

It is also important to discuss the observation that humiliation appeared to show the greatest bridge centrality among SD components across both networks. The central role of humiliation in our study is not surprising, as it is a multifaceted psychological construct that has long been recognized as a potent risk factor for the development and exacerbation of mental disorders (Kendler, Hettema, Butera, Gardner, & Prescott, 2003; McLoughlin, Sadath, McMahon, Kavalidou, & Malone, 2022). Moreover, the concept of repeated humiliation has been proposed to be the core aspect of the SD hypothesis (Selten & Ormel, 2023). Yet, the authors have not provided empirical evidence to support this hypothesis (Selten & Ormel, 2023). It can be hypothesized that repeated humiliation is closely related to low status, a psychosocial construct difficult to measure in the general population in a retrospective, self-descriptive way. In accordance with original accounts of the SD hypothesis (Selten & Ormel, 2023; Selten et al., 2020), we believe that repeated humiliation, rather than minority status itself, could be a significant indicator of an individual subordinate social position. Being part of a minority group can be an ambiguous experience, as it can be connected to the negative experience of being an outsider (Selten & Ormel, 2023). According to the SD hypothesis, it is needed to highlight that negative experiences associated with exclusion from the majority group, rather than representing the minority group itself, might initiate the development of psychosis (Selten, 2023). Indeed, some studies have demonstrated that minorities who do attain a sufficient level of social status may demonstrate a reduced risk of developing schizophrenia (Nimgaonkar et al., 2000; Suvisaari, Opler, Lindbohm, & Sallmén, 2014). These findings, together with those from our study, suggest that repeated experiences of humiliation, rather than minority affiliation alone, may be an important factor contributing to the development of PLEs. These considerations align with the original SD hypothesis of schizophrenia, which highlights prolonged, unwanted outsider status as a crucial element that disrupts dopamine activity, thereby contributing to the development of psychosis (Selten, 2023).

Increased sensitivity to momentary experiences of outsider status has been reported among individuals with first-episode psychosis (Reininghaus et al., 2016). In our study, almost one fourth of participants identified themselves as members of a minority group. However, we did not find any direct association of minority status with depressive and anxiety symptoms, AS, or PLEs. This observation indicates that the minority status requires the occurrence of other risk factors related to SD to trigger the onset of psychopathological symptoms, e.g. repeated humiliation. The relationship between long-term experience of a subordinate position status with psychosis has been widely discussed (Schalbroeck, 2023; Selten & Cantor-Graae, 2005; Selten & Ormel, 2023). One study revealed elevated rates of psychosis among indigenous ethnic groups that historically suffered poverty, trauma, and discrimination on a transgenerational level (Petrović-van der Deen et al., 2020). Another study demonstrated significantly higher odds of various psychiatric disorders (including psychotic disorders) in transgender individuals (Hanna et al., 2019), a minority experiencing numerous disparities compared to the general population, such as discrimination, economic hardship, and abuse (James et al., 2016).

One study revealed that individuals at risk for psychosis show a similar level of initial distress during social exclusion as healthy individuals, but they differ with respect to subsequent recovery (Lincoln, Johnson, Winters, & Laquidara, 2021). Specifically, this study revealed that increases in paranoid beliefs, negative emotions, and feelings of isolation persist for an extended time period after the exclusion experience in high-risk individuals. Our findings may be significant in understanding this phenomenon of maladaptive response to social stress, as cumulative experiences of humiliation and AS may contribute to their inability to cope effectively with the experience of social exclusion. Additionally, exposure to social exclusion has been associated with increased levels of negative emotions in individuals with chronic depression, compared to healthy controls (Jobst et al., 2015). This may be an important finding in the context of the ‘affective pathway’ of psychosis development (Myin-Germeys & Van Os, 2007). Moreover, our results are complementary to those reported by Toh et al. (2024), who revealed a relationship between humiliation and hallucination-like experiences. The authors found that the combination of cumulative humiliation and state anxiety might predict the occurrence of perceptual and cognitive disturbances (Toh et al., 2024). Interestingly, we used the same scale to assess humiliation in our study (Hartling & Luchetta, 1999).

Results of the present study must be interpreted considering several limitations. We did not evaluate our sample with clinical interviews designed to assess the presence of an underlying psychiatric diagnosis. However, there are reports suggesting that even self-reported PLEs, identified as false positive findings upon a thorough clinical assessment, might predict the development of psychosis (Kaymaz et al., 2012). Another point is that we were not able to record reasons of non-participation at baseline and thus insights into representativeness of the sample might be limited. However, it is needed to note that 70.2% of individuals responded to the invitation to participate in the survey. It is also important to note that non-completers had significantly higher levels of AS and PLEs. They also differed significantly in terms of sociodemographic characteristics (age, gender, education, and unemployment status). Some sociodemographic characteristics (i.e. education and unemployment status) were included as the SD components in our study. In this regard, this group might be perceived as the one with a greater vulnerability to develop clinically relevant psychopathology. This limitation might potentially explain as to why we did not replicate a mediating effect of depressive symptoms in the association between SD components and PLEs across both networks. Moreover, generalization of findings should be approached cautiously as individuals with a history of psychiatric treatment were excluded. Also, the conclusions about causality should be approached cautiously due to a relatively short observation period and a limited number of assessment time points. Furthermore, the minority status was self-assessed using only one item. It is further needed to note that there is no consensus about the inclusion of specific components within the SD concept. This is of particular importance for a network analysis, where addition or removal of specific variables might impact the results. Furthermore, observed coefficients were relatively small. Therefore, clinical relevance of observed associations might be limited. This might also be the consequence of limiting the study to participants with a negative history of psychiatric treatment and thus relatively low levels and variability across symptoms and specific psychological mechanisms.

Findings from the present study indicate that humiliation is the most central component within the SD hypothesis. It might be linked to the occurrence of PLEs through a mediating effect of AS. Altogether reported findings hold important implications from the perspective of public health and clinical practice. They suggest that therapeutic interventions focused on the experience of perceived humiliation might be relevant for individuals representing socially disadvantaged environments. Furthermore, our observations point to a further conceptualization of the SD concept by showing which aspects of this theory might be the most important for developing PLEs that serve as transdiagnostic markers of psychiatric disorders. However, in order to provide insights into causality, longitudinal studies with longer observation periods and multiple assessments are needed. Future studies should specifically focus on salience alterations in socially disadvantaged groups taking into consideration potential neurobiological mechanisms. Moreover, studies based on experimental approaches and ecological momentary assessment may offer further insights the relevance of SD hypothesis (Shovestul, Scharf, Liu, & Dodell-Feder, 2023).
